# MC5r and A2Ar Deficiencies During Experimental Autoimmune Uveitis Identifies Distinct T cell Polarization Programs and a Biphasic Regulatory Response

**DOI:** 10.1038/srep37790

**Published:** 2016-11-25

**Authors:** Darren J. Lee, Janine Preble, Stacey Lee, C. Stephen Foster, Andrew W. Taylor

**Affiliations:** 1 Department of Ophthalmology/Dean McGee Eye Institute, University of Oklahoma Health Sciences Center, Oklahoma City, Oklahoma, USA; 2 Massachusetts Eye Research and Surgery Institute, Waltham, Massachusetts, USA; 3 Ocular Immunology and Uveitis Foundation, Waltham, Massachusetts, USA; 4 Harvard Medical School, Boston, Massachusetts, USA; 5 Department of Ophthalmology, Boston University School of Medicine, Boston, Massachusetts, USA

## Abstract

Autoantigen-specific regulatory immunity emerges in the spleen of mice recovering from experimental autoimmune uveitis (EAU), a murine model for human autoimmune uveoretinitis. This regulatory immunity provides induced tolerance to ocular autoantigen, and requires melanocortin 5 receptor (MC5r) expression on antigen presenting cells with adenosine 2 A receptor (A2Ar) expression on T cells. During EAU it is not well understood what roles MC5r and A2Ar have on promoting regulatory immunity. Cytokine profile analysis during EAU revealed MC5r and A2Ar each mediate distinct T cell responses, and are responsible for a functional regulatory immune response in the spleen. A2Ar stimulation at EAU onset did not augment this regulatory response, nor bypass the MC5r requirement to induce regulatory immunity. The importance of this pathway in human autoimmune uveitis was assayed. PBMC from uveitis patients were assayed for MC5r expression on monocytes and A2Ar on T cells, and comparison between uveitis patients and healthy controls had no significant difference. The importance for MC5r and A2Ar expression in EAU to promote the induction of protective regulatory immunity, and the expression of MC5r and A2Ar on human immune cells, suggests that it may be possible to utilize the melanocortin-adenosinergic pathways to induce protective immunity in uveitic patients.

Uveitis is the third leading cause of blindness among Americans with an incidence of 25.6–122 cases per 100,000 persons per year and a prevalence of 69–623 per 100,000 persons^1^,^2^,^3^. Each year 17.6% of active uveitis patients experience a transient or permanent loss of vision, and 12.5% will develop glaucoma^4^. After an initial episode of anterior uveitis 33% will experience three or more recurrences within 5 years^5^. The mechanisms contributing to the relapsing and remitting nature of chronic autoimmune uveitis, and how to achieve prolonged remission is unknown.

In order to better understand the immunobiology of autoimmune uveitis, experimental autoimmune uveitis (EAU), a mouse model of human autoimmune uveitis is used^6^,^7^,^8^. Inflammation in C57BL/6 J mice resolves at 75–90 days, at which time post-EAU regulatory immunity is found in the spleen^9^. An important role of post-EAU regulatory immunity is to prevent a memory immune response to ocular auto-antigen, and can be transferred to suppress EAU in recipient mice^9^,^10^,^11^,^12^. Mice that have recovered from EAU and have regulatory immunity show a naïve immune response when reimmunized for EAU^13^. The induction of this post-EAU regulatory immunity is dependent on the eye since enucleation prior to induction of EAU prevents the emergence of regulatory immunity in the spleen^9^. However, it is likely that this is a mechanism utilized by immune privileged sites in the body to prevent a second memory immune response from occurring. As such, regulatory immunity can also be found in the spleen of mice that have recovered from the mouse model of multiple sclerosis, experimental autoimmune encephalomyelitis^14^.

Post-EAU regulatory immunity is also dependent on the expression of the Melanocortin 5 receptor (MC5r)^10^,^11^,^13^, one of the melanocortin receptors for the immunomodulating neuropeptide, alpha-melanocyte stimulating hormone (α-MSH)^15^,^16^. The neuropeptide, α-MSH, is constitutively expressed in the healthy ocular microenvironment, and inhibits macrophages and dendritic cell pro-inflammatory signaling and function^17^,^18^. We have demonstrated that α-MSH treatment of EAU is effective in reducing ocular inflammation^19^,^20^ and promotes expansion of the regulatory APC in the post-EAU spleen^11^. These regulatory APC have been identified as CD11b^+^F4/80^+^Ly-6G^+^Ly-6C^low^, and have up-regulated the ectoenzymes, CD39 and CD73, to generate adenosine^11^. The increased concentration of local adenosine promotes the emergence of post-EAU Treg cells in the spleen through stimulation of adenosine 2 A receptor (A2Ar) on the T cell^10^,^11^ *in vivo*, and *in vitro*^21^,^22^. In addition, targeted stimulation of A2Ar during the peak of EAU promotes resolution of disease, and is marked by an induction of regulatory T cell activity^11^. These observations demonstrate that the induction of post-EAU regulatory immunity that provides resistance to EAU occurs through a linear pathway that requires MC5r expression on a CD11b^+^F4/80^+^CD39^+^CD73^+^Ly-6C^hi^Ly-6G^+^ macrophage that promotes Treg cells through A2Ar stimulation^11^,^12^.

The current treatment paradigm for autoimmune uveitis is to suppress the inflammation for a period of time that is long enough for the eye or the immune system to re-establish regulatory immunity to ocular autoantigen on its own^23^,^24^. This immunosuppressive strategy places the patient at an increased susceptibility to infection. In addition, non-steroidal anti-inflammatory drugs such as naproxen and celecoxib can cause gastrointestinal bleeding^24^,^25^. Biologics are another class of therapies that target specific cytokines or cytokine receptors to inhibit the inflammatory response^26^,^27^,^28^. However, because these are relatively new treatments for uveitis it is not yet known if these will result in sustained remission of uveitis. The long-term safety of biologics is unknown, and can carry a high economic burden^29^. Therefore, better treatments for autoimmune uveitis are necessary. Moreover, treatments that target pathways to promote regulatory immunity to provide protection from relapse would have the highest impact.

Our previous studies with EAU mice have revealed an important link between the immunomodulating melanocortin and adenosinergic pathways through the expression of MC5r on APC, and A2Ar on lymphocytes^10^,^11^,^12^. How the systemic immunobiology of EAU recovery is affected through a MC5r or A2Ar deficiency has not been explored nor has it been explored if these receptors are expressed in uveitis patients. In this study we show that MC5r and A2Ar have distinct roles in the recovery from EAU, and that MC5r expression on specific subsets of monocytes and A2Ar expression on CD4 T cells is similar between uveitis patients and healthy controls.

## Results

### The systemic cytokine profile during EAU is distinct for MC5r and A2Ar deficient mice

**Figure 1:**
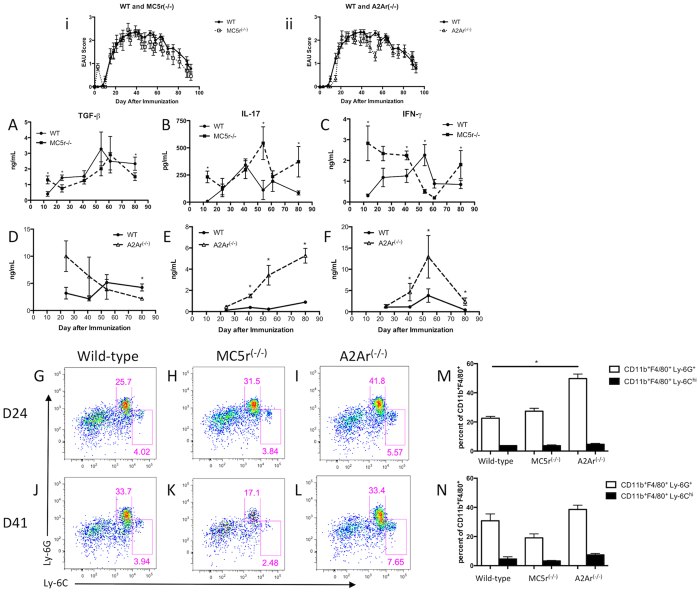
Time course of cytokine profile and macrophage subsets in the spleen during EAU. Shown at the top are mean EAU scores ± SEM of wild-type and MC5r^(−/−)^ mice (i) and wild-type and A2Ar^(−/−)^ mice (ii). The spleen was collected at different time points during the course of disease from wild-type, MC5r^(−/−)^, and A2Ar^(−/−)^ mice, spleen cells were re-activated *in vitro* with IRBP peptide, and supernatants were analyzed for TGF-β, IL-17, and IFN-γ (**A**–**F**). Each time point is an average ± SEM of 5–10 mice collected from at least three different experiments. Spleen cells were analyzed by flow cytometry following staining for CD11b, F4/80, Ly-6C, and Ly-6G (**G**–**L**). They were first gated on CD11b^+^F4/80^+^ cells, and are representative of four independent experiments. The mean and SEM for the four experiments are shown for Day 24 (M) and Day 41 (N). Statistical significance (P ≤ 0.05) is designated by *.

Since the induction of Tregs that provide resistance to EAU require MC5r expression on the APC, and A2Ar expression on T cells^10^,^13^, is the T cell response in the spleen altered during the course of EAU? C57BL/6 J, MC5r^(−/−)^, and A2Ar^(−/−)^ mice were immunized to induce EAU. As shown before and in Fig. 1, the course of disease between wild-type, MC5r^(−/−)^, and A2Ar^(−/−)^ mice was not significantly different (Fig. 1i, 1ii)^10^,^11^. The onset of disease was between days 15–24, a chronic phase occurred between days 25–62, and resolution of disease occurred from days 63–80. Spleens from wild-type, MC5r^(−/−)^, and A2Ar^(−/−)^ mice were collected during the onset (day 24), chronic phase (day 41), just before resolution phase (day 54), and post-EAU (day 80). In order to study an antigen-specific T cell response, the spleen cells were reactivated with human interphotoreceptor retinoid binding protein (IRBP) peptide (amino acids 1–20) for two days, and supernatants were assayed for IFN-γ, TGF-β, and IL-17. There was a gradual increase in TGF-β production with a peak just before resolution of EAU (Fig. 1A,D). The production of IL-17 increased until the chronic phase of disease, and then gradually decreased (Fig. 1B,E). The concentration of IFN-γ increased until resolution began and quickly decreased (Fig. 1C,F). In MC5r^(−/−)^ mice TGF-β production was similar to wild-type EAU mice, but was significantly lower at the end of disease (Fig. 1A), while IL-17 continued to increase through disease, and was significantly more at the end of disease (Fig. 1B). There was a biphasic production of IFN-γ, which was significantly higher at the start of EAU in MC5r^(−/−)^ mice; however its production decreased through the chronic phase, and then significantly increased during resolution of EAU (Fig. 1C). In contrast, A2Ar^(−/−)^ mice showed a steady decline in TGF-β production (Fig. 1D), a steady increase in IL-17 production (Fig. 1E), and an increase in IFN-γ until resolution when it decreased but was still significantly higher than wild-type at the end of disease (Fig. 1F). These observations showed distinct cytokine profiles that occurred during EAU with either a MC5r or an A2Ar deficiency. At resolution of EAU, while the wild type mice can be shown to have a Treg cell response, the A2Ar deficient mice have a Th17 dominant T cell response, and the MC5r deficient mice have a Th1 dominant response to IRBP peptide in the spleen.

In wild type EAU mice the post-EAU Tregs require activation by a post-EAU suppressor CD11b^+^F4/80^+^ APC that was Ly-6C^lo^Ly-6G^+^ (Ly-6G^+^ APC), but not by a more conventional CD11b^+^F4/80^+^ APC expressing Ly-6C^hi^Ly-6G^−^ (Ly-6C^hi^ APC)^11^. The post-EAU spleens from MC5r^(−/−)^ mice have significantly less Ly-6G^+^ APC^11^. Therefore, there may be differences in the Ly-6G^+^ and Ly-6C^hi^ populations of APC at the onset, and during the chronic phase of EAU to account for the changes in T cell activity in the spleen. The Ly-6C^hi^ population showed no change at the onset compared to the chronic phase, but a slight increase in the Ly-6G^+^ population was observed (Fig. 1G,J). In MC5r^(−/−)^ mice the Ly-6C^hi^ APC showed a slight decrease and a two-fold decrease in the Ly-6G^+^ APC in the chronic phase compared to the onset (Fig. 1H,K). The A2Ar^(−/−)^ mice had slightly more Ly-6C^hi^ APC and Ly-6G^+^ APC compared to wild-type and MC5r^(−/−)^ mice at the onset of disease (Fig. 1I,L). At the chronic phase of disease the A2Ar^(−/−)^ mice had more of the Ly-6C^hi^ APC compared with the onset, and in comparison to wild-type and MC5r^(−/−)^ mice at the chronic phase of disease. While the Ly-6G^+^ APC in the A2Ar^(−/−)^ mice at the chronic phase is less than the onset, it was similar in percentage compared to wild-type at the chronic phase (Fig. 1I,L,J). Taken together, these observations demonstrate that knocking out MC5r or A2Ar had very distinct effects on the cytokine profile, and the distribution of cell populations in the spleen during the course of EAU.

### There is a biphasic systemic regulatory response during the course of EAU

**Figure 2:**
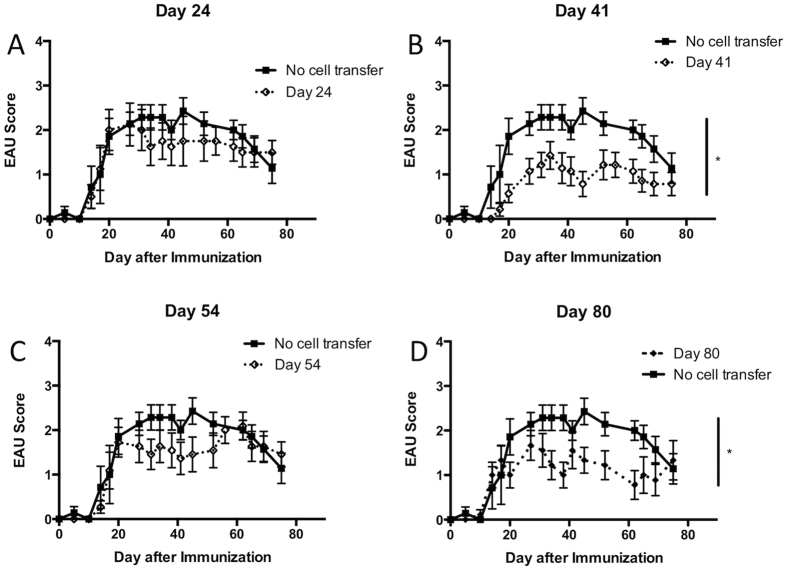
Assay for functional regulatory activity in the spleen of EAU mice. Spleens from mice immunized for EAU were collected at the onset (day 24), chronic phase (day 41), before resolution (day 54), and post-EAU (day 80). Spleen cells were re-activated *in vitro* with IRBP peptide and transferred to recipient mice immunized for EAU. The mice were clinically scored for observable symptoms of uveitis. Shown are mean EAU scores ± SEM of each group of mice each day. There are groups of mice that did not receive spleen cells (solid line) and recipient mice (dashed line) day 24 (**A**), day 41 (**B**), day 54 (**C**), and day 80 (**D**). Each group was repeated three times and represents 8–12 recipient mice per group. Statistical significance (P ≤ 0.05) is designated by *.

In order to determine when functional regulatory immunity that suppresses EAU emerges in the spleen, spleen cells from EAU mice at different phases of EAU were adoptively transferred into to recipient mice immunized to induce EAU. Spleen cells were collected at the onset of EAU (day 24), during the chronic phase (day 41), just before resolution (day 54), and post-EAU (day 80). Spleen cells were reactivated *in vitro* for 48 hours with IRBP peptide, then injected into recipient mice immunized to induce EAU. Mice that received naïve spleen cells showed no change in course of disease compared to mice that received no cell transfer (Supplementary Fig. 2A). Mice that received spleen cells from the onset of EAU or just before resolution showed no difference in EAU duration or severity (Fig. 2A,C, Supplementary Fig. 2B,D). In contrast, during the chronic phase we observed a potent regulatory response to prevent EAU in recipient mice (Fig. 2B, Supplementary Fig. 2C), and as expected, mice that received post-EAU spleen cells showed significant suppression of disease (Fig. 2D, Supplementary Fig. 2E). These observations demonstrate a novel biphasic regulatory response that occurs in the spleen of mice during the chronic phase of disease present at day 41 but not at day 54 and then returns to the post-EAU spleen. Day 41 also corresponds to the highest levels of Th17 cells in the spleen of EAU mice (Fig. 1B).

**Figure 3:**
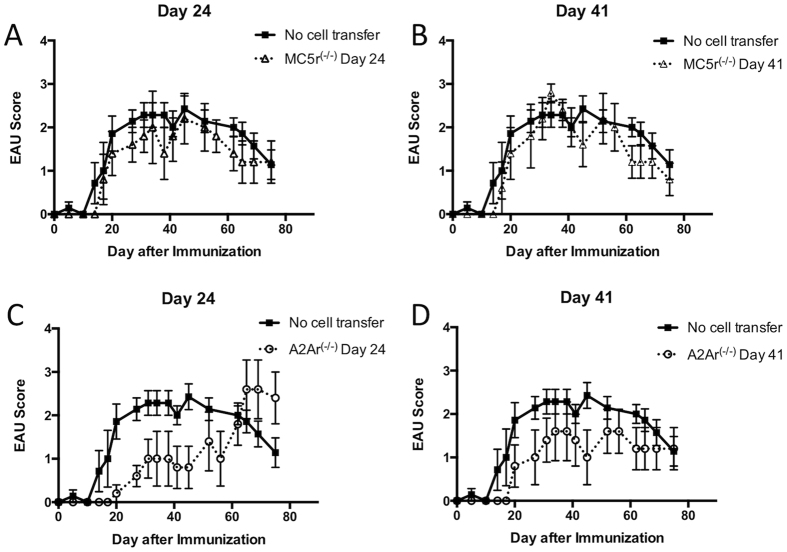
Assay for functional regulatory activity in the spleen of MC5r^(−/−)^ and A2Ar^(−/−)^ EAU mice. Spleens from MC5r^(−/−)^ and A2Ar^(−/−)^ mice immunized for EAU were collected at the onset (day 24) and chronic phase (day 41) of EAU. Spleen cells were re-activated *in vitro* with IRBP peptide and transferred to recipient mice immunized for EAU. Shown are mean EAU scores ± SEM of each group of mice each day. The mice groups are mice that did not receive spleen cells (solid line), and the mice that did (dashed line) receive spleen cells from MC5r^(−/−)^ mice at day 24 (**A**), or day 41 (**B**), or mice that received spleen cells from A2Ar^(−/−)^ mice at day 24 (**C**), or day 41 (**D**). Each experiment was repeated twice and represents 6–8 recipient mice per group. Statistical significance (P ≤ 0.05) is designated by *.

To see if the regulatory immunity in the spleen at the chronic phase of EAU is also present in MC5r^(−/−)^ or A2Ar^(−/−)^ mice, spleen cells were collected at the onset and during the chronic phase of EAU from MC5r^(−/−)^ or A2Ar^(−/−)^ EAU mice. The spleen cells were reactivated *in vitro*, and transferred to recipient mice that were immunized for EAU. Spleen cells from MC5r^(−/−)^ mice had no suppressive capacity at the onset or chronic phase of disease (Fig. 3A,B). Mice that received spleen cells from A2Ar^(−/−)^ mice at the onset showed a delay in disease, but no statistical significance was observed when compared with mice that received no spleen cells Fig. 3C) or with mice that received spleen cells from the wild type mice at the onset of disease (Supplementary Fig. 3A). In addition, the spleen cells from A2Ar^(−/−)^ mice at the chronic phase of disease had no significant suppressive capacity when compared to mice that received no spleen cells (Fig. 3D) but were also not significantly different from spleen cells from A2Ar competent mice during the chronic phase (Supplementary Fig. 3B). These observations show that the biphasic regulatory response in the spleen requires expression of A2Ar and MC5r.

### A2Ar stimulation during the onset of EAU requires MC5r to suppresses inflammation

**Figure 4:**
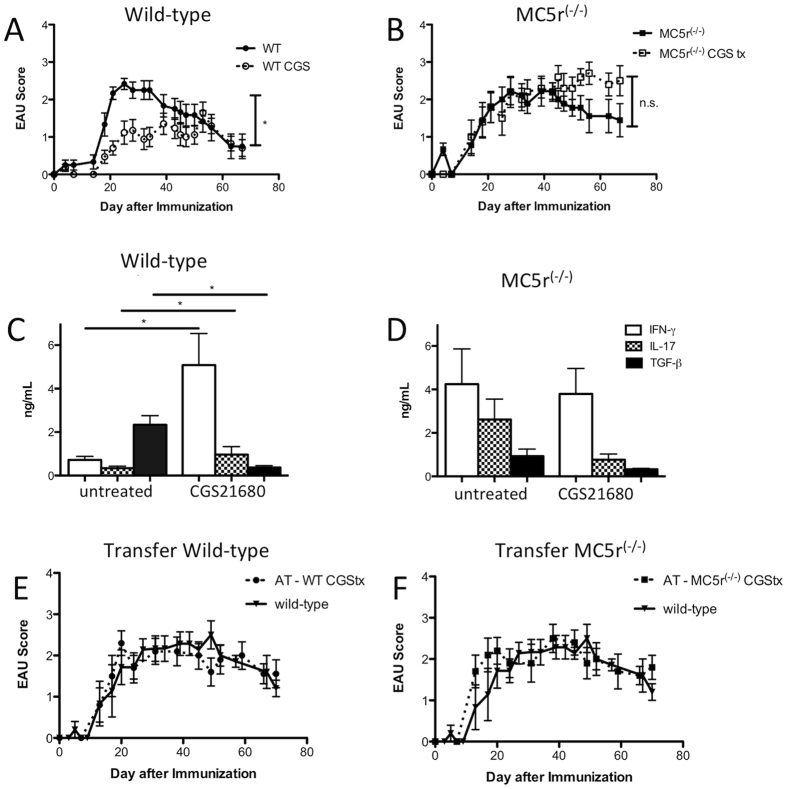
Effect of A2Ar stimulation on EAU and induction of regulatory immunity. Shown are mean EAU scores ± SEM. Mice immunized for EAU were treated with CGS21680 (dashed line, n = 17) at the onset of EAU (day 17, 18, 19) and compared with untreated mice immunized for EAU (solid line, n = 12) (**A**). MC5r^(−/−)^ mice were immunized for EAU and treated at the onset of EAU with CGS21680 (dashed line, n = 10) and compared with untreated MC5r^(−/−)^ mice immunized for EAU (solid line, n = 9) (**B**). Spleens were collected at the resolution of EAU from wild-type mice that were either untreated (n = 7–13) or CGS21680 treated (n = 5,6); and MC5r^(−/−)^ mice that were untreated (n = 7–13) or CGS21680 treated (n = 8–10). The spleen cells were re-activated *in vitro* with IRBP peptide for 48 hours, and the supernatants were assayed for IFN-γ, IL-17, and TGF-β (**C,D**). Bar graphs represent mean ± SEM of the indicated cytokine concentration. Recipient mice immunized for EAU received the re-activated spleen cells from wild-type (n = 10) or MC5r^(−/−)^ (n = 10) mice treated with CGS21680 at the onset of disease, and the retina was observed for signs of inflammation (**E,F**). Statistical significance (P ≤ 0.05) is designated by *.

CGS21680 is the only commercially available high affinity A2Ar agonist and has been shown to have specificity for A2Ar and not the other adenosine receptors on T cells^30^. CGS21680 given to EAU mice at the peak of disease is effective in accelerating resolution of EAU and inducing regulatory immunity in the spleen^11^. We asked if the stimulation of A2Ar at the onset of EAU is just as effective as treating at the peak of disease. Mice immunized for EAU given three systemic injections of CGS21680 on days 17, 18, and 19 showed significantly suppressed EAU scores (Fig. 4A). It has been previously demonstrated that melanocortin receptor stimulation by alpha-melanocyte stimulating hormone (α-MSH) is effective in suppressing EAU and inducing regulatory immunity in the spleen, but requires expression of MC5r and A2Ar^10^,^11^,^12^. Therefore, we asked if MC5r is necessary for suppression of EAU with treatment of CGS21680 at the onset of disease. CGS21680 treatment of MC5r^(−/−)^ EAU mice did not suppress the course of EAU (Fig. 4B). This showed that MC5r expression is necessary for CGS21680 treatment of EAU at the onset of disease.

### A2Ar stimulation during the onset of EAU does not induce regulatory immunity

We next asked if CGS21680 given at the onset of EAU also induced regulatory immunity in the spleen. The cytokine profile of treated mice compared with untreated mice showed significantly higher IFN-γ, and IL-17, with significantly lower TGF-β (Fig. 4C). As expected, the cytokine profile in the spleen of post-EAU MC5r^(−/−)^ mice showed no difference from the untreated post-EAU MC5r^(−/−)^ mice (Fig. 4D). We also assessed if the spleens had functional regulatory immunity by adoptive transfer of reactivated spleen cells to recipient mice immunized for EAU. Neither recipient mice that received spleen cells from CGS21680 treated wild-type or CGS21680 treated MC5r^(−/−)^ mice showed suppression of EAU (Fig. 4E,F), compared to mice that received post-EAU spleen cells (Fig. 2D). These observations showed that A2Ar stimulation at the onset of EAU is effective in suppressing disease, but does not promote regulatory immunity that provides resistance to EAU.

### Expression of MC5r and A2Ar is similar between uveitis patients and healthy volunteers

**Figure 5:**
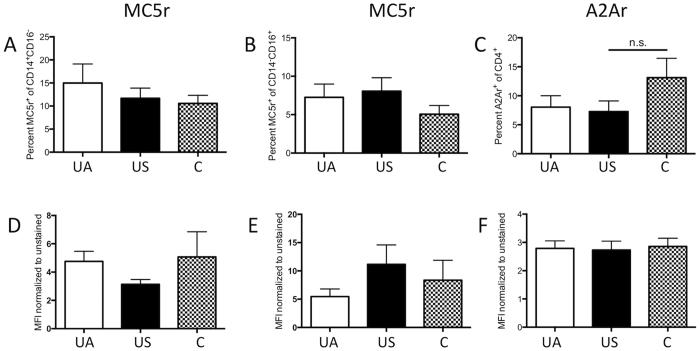
Expression of MC5r and A2Ar on PBMC subsets from uveitis patients. Shown are bar graphs of the mean ± SEM of the percentage of cells that express the indicated receptor. Whole blood was collected from non-infectious uveitis patients or non-uveitis controls (C, n = 11). Uveitis patients were divided into two groups, an active group (UA) defined as having inflammation within a year from the time of the blood draw (n = 20), and a suppressed group (US) that had no inflammation for more than a year from the time of the blood draw (n = 20). PBMCs were isolated and stained for CD14, CD16, CD4, MC5r and A2Ar. Shown is the percentage of CD14^+^CD16^−^ cells that are MC5r^+^ (**A**), percentage of CD14^−^CD16^+^ cells that express MC5r (**B**), and percentage of CD4^+^ cells that express A2Ar. The mean fluorescence intensity (MFI) normalized to unstained cells is shown (**D–F**). No statistical significance is designated by n.s.

The relapsing and remitting ocular inflammation experienced by chronic autoimmune uveitis patients indicates that the mechanisms of ocular immune privilege can resolve the inflammation, but does not promote regulatory immunity that can prevent the relapse. Therefore, immune cells from patients with chronic uveitis were assayed for MC5r and A2Ar expression to see if there are changes in the receptor expression associated with the chronic nature of autoimmune uveitis. Peripheral blood mononuclear cells (PBMC) were collected from healthy volunteers, uveitis patients with symptoms of uveitis within a year of the blood draw (active), and uveitis patients without symptoms of uveitis for more than a year (suppressed). We chose the one year mark to distinguish the patient cohorts because patients being treated with alkylating agents tend to achieve durable remission if the treatment is continued for at least one year^31^. The total number of patients from each group with the type of uveitis and the type of treatment at the time of collection is shown in Table 1 and Table 2. The types of uveitis were relatively similar between the active and suppressed groups with anterior uveitis as the most common type of uveitis (5/20 for active and 7/20 for suppressed, Table 1). The treatment that patients were taking showed more patients in the active group on steroids (4/20 active compared to 0/20 in the suppressed group, Table 2), this is because these patients had active uveitis and required immediate suppression of the uveitis, there were also two patients in the suppressed group that were not on any treatment. With the exception of the steroid patients and those off all medications the type of treatments were relatively similar between the two uveitis cohorts. PBMC were stained on the day they were collected for CD4, CD14, CD16, A2Ar, and MC5r, and analyzed by flow cytometry. We assessed MC5r expression on CD14^+^CD16^−^ and CD14^−^CD16^+^ monocyte sub-populations and A2Ar expression on CD4 T cells (Fig. 5). The percentage of monocytes that expressed MC5r and mean fluorescence intensity (MFI) of MC5r was no different between active, suppressed, and healthy population (Fig. 5A,B,D,E). A2Ar expression trended lower in the suppressed population but was not significantly different between any of the three groups, and the MFI of A2Ar was not different between the three groups (Fig. 5C,F). The number of patients with active uveitis at the time of collection was 11 out of the 20 total from the active group and the receptor expression was not significantly different compared with the other active group of patients (Supplementary Fig. 4). MC5r expression on classical macrophages trended higher in the suppressed group, but was not significantly different compared to the active and control groups. There was also no significant difference in receptor expression when compared to age, current treatment, or the type of uveitis the patient had (data not shown). These observations demonstrate that chronic autoimmune uveitis is not due to a deficiency in MC5r or A2Ar expression, and suggests it may be possible to suppress uveitis and induce regulatory immunity by stimulating the melanocortin-adenosinergic pathway in uveitis patients.

## Discussion

There were separate and distinct effects of A2Ar and MC5r expression on T cell polarization in the spleen during the course of EAU. The treatment of EAU with an A2Ar agonist did not induce regulatory immunity when treated at the onset of disease, and the accelerated resolution of EAU by the agonist required expression of MC5r. Importantly, stimulation of the melanocortin-adenosinergic pathway may be possible in human uveitis patients because expression of MC5r and A2Ar was similar between uveitis patients and controls.

Since MC5r is necessary on the CD11b^+^ macrophage, and A2Ar is required on the Treg cell for the induction of protective regulatory immunity after EAU^11^, what the role these receptors have on the systemic immune response during the course of EAU was assayed. The cytokine profile in the spleen indicates that the onset of disease begins with a Th1 T cell response, the chronic phase of disease correlates with a Th17 T cell response, and as EAU resolves a dominant Treg response emerges. These observations show that MC5r and A2Ar are necessary for the emergence of the Treg response in the spleen at resolution of EAU. Interestingly, a deficiency in MC5r results in a dominant Th1 response at resolution, and the A2Ar deficiency results in a dominant Th17 response at resolution. This may be explained by the possibility of differential expression of the receptors by immune cell subtypes^10^,^11^. In addition, these distinct responses may reflect the heterogeneity of uveitis in the human population^32^.

We previously described a linear pathway of the induction of post-EAU regulatory immunity that requires MC5r expression on a CD11b^+^F4/80^+^CD39^+^CD73^+^Ly-6C^hi^Ly-6G^+^ macrophage that promotes Treg cells through A2Ar stimulation^11^,^12^. Further support for the linear pathway is demonstrated with the observation that induction of post-EAU regulatory immunity through α-MSH treatment of EAU mice requires A2Ar expression^11^. However, our new observation showed that this pathway may not be linear. The observation that A2Ar stimulation does not induce post-EAU regulatory immunity showed that the resolution in the eye is necessary for induction of this regulatory immunity. Moreover, treatment of MC5r^(−/−)^ mice with the A2Ar agonist was ineffective in suppressing EAU, and did not promote regulatory immunity. This suggests that there may be some cross talk between the melanocortin and adenosinergic pathway through the Treg cells and APC for post-EAU protective regulatory immunity, as has been discussed in the literature^33^,^34^.

Another interesting observation in this study is the biphasic regulatory immunity observed in the spleen. Because we see the potent regulatory activity at the chronic phase, but not just before resolution (day 54) this is an indication that regulatory activity emerges during the chronic phase, then leaves or wans in the potency to then re-emerge at resolution. Since the EAU does not resolve until after the chronic phase, this suggests that the observed uveitis in the eye lags behind the systemic immune response. Moreover, this indicates that the resolution of uveitis occurs independently of the induction of systemic regulatory immunity that provides resistance to uveitis. In addition, our observations demonstrate that MC5r and A2Ar are not required for the resolution of uveitis, but are necessary for the induction of post-EAU regulatory immunity in the spleen, and have distinct roles in the systemic immune response found in the spleen^9^,^11^.

In humans, the classical macrophage population (CD14^+^CD16^−^) population showed a slightly higher percentage of MC5r^+^ monocytes compared to the alternatively activated (CD14^−^CD16^+^) monocytes. A previous study found the CD14^+^ monocytes did not express MC5r mRNA^35^. This discrepancy may suggest that MC5r is stably expressed on the cell surface. It remains to be seen which population is sensitive to α-MSH stimulation, and which population is regulatory in function. Furthermore, it has been observed in the mouse model of multiple sclerosis that the Ly-6C^hi^ population can change from inflammatory to regulatory during the course of disease^36^,^37^, so additional investigation of these two populations is necessary to further determine the functionality of each and if it changes depending on the status of the uveitis.

Comparison of the MC5r expression on the subpopulations of monocytes from active uveitis patients, suppressed uveitis patients, and healthy patients showed that there was no significant difference between each of the patient populations. In addition we observed no difference between these populations when we examined A2Ar expression on the CD4^+^ T cell population. This is an important discovery because it indicates a potential to stimulate these receptors as a novel treatment for autoimmune uveitis. This sort of treatment is novel because it both suppresses the inflammatory response while simultaneously promoting a regulatory immune response^22^,^38^,^39^, and is independent of corticosteroid-driven pathways^40^.

The observation that A2Ar stimulation is effective in suppressing EAU, but does not promote regulatory immunity demonstrates that the resolution of ocular inflammation is not dependent on the emergence of systemic regulatory immunity. Patients with chronic autoimmune uveitis are treated until the ocular inflammation is resolved, but it often relapses^5^. The results of this manuscript suggest that the timing may be important to consider with respect to the induction of regulatory immunity that resists relapse.

The expression of MC5r and A2Ar was not different in either uveitis populations compared to the controls. It is still possible that there are polymorphisms in MC5r or A2Ar, or a block in downstream signaling pathways that could prevent their stimulation from mediating regulatory immunity. In order to address this possibility, additional studies are needed to assess receptor functionality. However, because these receptors are present on a similar percentage of cells as the controls, it implies that maybe it is possible to stimulate the melanocortin adenosinergic pathway as a potential treatment for chronic autoimmune uveitis. An *in vivo* therapy would involve the use of pharmaceutical MC5r and A2Ar agonists to stimulate both pathways. Also, there is the potential for an *ex vivo* therapy using the approach similar to the completed phase I clinical trial that injected patients with Type 1 diabetes with their own polyclonal Tregs purified from PBMC and expanded *in vitro*^41^,^42^. In mice this pathway is spontaneously activated, presumably as ocular immune privilege is re-established^43^,^44^,^45^,^46^, and in humans with chronic autoimmune uveitis it may need a boost.

## Methods

### Mice

All mouse procedures described in this study were approved by the Boston University Institutional Animal Care and Use Committee (BU IACUC) and all mouse study methods were carried out in accordance with the relevant guidelines approved by the BU IACUC. C57BL/6 J mice were purchased from Jackson Laboratories, A2Ar^(−/−)^ mice were obtained from Dr. Jiang-Fen Chen (Boston University School of Medicine, Boston, MA). The MC5r^(−/−)^ mice were obtained from Roger D. Cone (Oregon Health Sciences, Portland, Oregon). Both A2Ar^(−/−)^ mice and MC5r^(−/−)^ mice were housed and bred in the Boston University Laboratory Animal Science Center.

### Experimental Autoimmune Uveoretinitis (EAU)

Mice were immunized for EAU as previously described^12^. Briefly, an emulsion of complete Freund’s adjuvant (CFA) with 5 mg/mL desiccated *M. tuberculosis* (Difco Laboratories, Detroit, MI) and 2 mg/ml human interphotoreceptor retinoid binding protein (IRBP) peptide (amino acids 1–20) (Genscript, Piscataway, NJ) was used to immunize mice for EAU. A volume of 100 μL of the emulsion was injected subcutaneously at two sites in the lower back followed by an intraperitoneal injection of 0.3 μg pertussis toxin. The severity of retinal inflammation during the course of EAU was evaluated every 3–4 days by fundus examination using a slit lamp microscope. Before examining the retina, the iris was dilated with 1% tropicamide, the cornea was numbed with 0.5% proparacaine, and the cornea was flattened with a glass coverslip in order to examine the retina. The clinical signs of observable infiltration and vasculitis in the retina were scored on a 5 point scale as previously described^47^. Both eyes were scored and the higher score was used to represent that mouse for that day, the average score for the group of mice was then calculated.

### CGS21680 treatment

The A2Ar agonist, CGS21680 (Tocris, Bristol, U.K.) in DMSO (27.7 mg/mL) was diluted in PBS to a final concentration of 0.05 mg/mL. Mice received CGS21680 at 0.5 mg/kg given i.p. once a day for three days.

### *In Vitro* Stimulation

Spleens were collected into 5% FBS in RPMI supplemented with 10 μg/ml Gentamycin (Sigma), 10 mM HEPES, 1 mM Sodium Pyruvate (BioWhittaker), Nonessential Amino Acids 0.2% (BioWhittaker) and made into a single cell suspension that was depleted of red blood cells using RBC lysis buffer (Sigma, St Louis, MO). The spleen cells were resuspended in serum free media (SFM) and IRBP peptide was added at 50 μg/mL for 48 hours at 37 °C and 5% CO_2_ to reactivate antigen specific T cells. SFM consisted of RPMI-1640 with 1% ITS + 1 solution (Sigma) and 0.1% BSA (Sigma). Following the *in vitro* reactivation supernatants were collected and analyzed and/or cells were collected for adoptive transfer into recipient mice. Recipient mice were immunized for EAU on the same day as receiving 1 × 10^6^ reactivated spleen cells.

### Cytokine Assays

Cell culture supernatants were assayed for IL-17, IFN–γ, and TGF–β. The concentration of IFN–γ in the supernatant was measured by sandwich ELISA (IFN-γ detection and biotinylated IFN-γ antibodies from BD Biosciences). The TGF–β concentration was measured with the standard Mv1Lu bioassay^48^. The IL-17 concentration in the supernatant was measured using an IL-17 ELISA kit (R&D systems).

### Human Studies

Institutional Review Board Approval was obtained from New England IRB, the University of Oklahoma Human Research Participant Protection IRB, and the IRB of Boston University Medical Campus was obtained to collect and analyze PBMC from human uveitis patients. All experiments involving human subject were carried out in accordance with the relevant guidelines approved by the appropriate IRB. Prior to the collection of patient samples informed consent was obtained from all subjects. Patient samples were de-identified and limited patient information was made available to the research staff.

### Peripheral Blood Mononuclear Cell (PBMC) Preparation

Whole blood was collected from patients by the clinical staff at MERSI or DMEI. Within 24 hours from the time of collection the blood was processed using SepMate 50 tubes (Stemcell Technologies, Vancouver, BC, Canada) to isolate the PBMCs from the blood. PBMCs were then immediately stained with antibodies for flow cytometry analysis.

### Flow Cytometry

Mouse spleen cells were washed with PBS with 1% BSA (staining buffer), blocked with mouse IgG in staining buffer, then stained with conjugated antibodies. Antibodies used were anti-CD11b (clone M1/70, Biolegend, San Diego, CA), anti-Ly-6C (clone HK1.4, Biolegend), anti-Ly-6G (clone 1A8, Biolegend), and F4/80 (clone BM8, eBiosciences, San Diego, CA).

Human PBMCs were stained with anti-CD14 (clone M5E2, Biolegend), anti-CD16 (clone 3G8, Biolegend), anti-CD4 (clone OKT4, Biolegend), anti-MC5r (polyclonal goat, Santa Cruz Biotechnology, Dallas, TX), anti-A2Ar (clone 5H30, Santa Cruz Biotechnology), and anti-goat conjugated to allophycocyanin (Jackson ImmunoResearch Laboratories, West Grove, PA). Prior to anti-A2Ar staining, the cells were fixed and permeabilized.

Stained cells were analyzed in the Boston University Flow Cytometry Core Facility on a BD LSRII (BD Biosciences) and data was analyzed using FlowJo Software (Tree Star, Inc., Ashland, OR).

### Statistics

Statistical significance between EAU scores was determined using nonparametric Mann-Whitney U test between groups of mice. Two-way ANOVA was also used to assess significant changes in the tempo of disease between the groups of treated EAU mice. Cytokine concentrations were statistically analyzed by one-way ANOVA with post-test Bonferroni comparison analysis. Statistical significance was determined when P ≤ 0.05.

## Additional Information

**How to cite this article**: Lee, D. J. *et al.* MC5r and A2Ar Deficiencies During Experimental Autoimmune Uveitis Identifies Distinct T cell Polarization Programs and a Biphasic Regulatory Response. *Sci. Rep.* **6**, 37790; doi: 10.1038/srep37790 (2016).

**Publisher's note:** Springer Nature remains neutral with regard to jurisdictional claims in published maps and institutional affiliations.

## Supplementary Material

Supplementary Information

## Figures and Tables

**Table 1:** Type of Uveitis.

	UA	US
Anterior	5	7
Posterior	4	2
Intermediate	1	2
Panuveitis	3	2
JIA/JRA/RA	2	1
HLA-B27	3	2
Scleritis	2	2
Behcet’s	0	2

**Table 2:** Current Treatment.

	UA	US
Steroid	4	0
NSAID	2	3
IMT*	6	5
Biologic^†^	1	2
Steroid, IMT	1	0
Biologic, IMT	6	8
None	0	2

^*^IMT – immunomodulatory therapy.

^†^Biologic – anti-TNF therapy.
