# Supplementary material for: MC5r and A2Ar Deficiencies During Experimental Autoimmune Uveitis Identifies Distinct T cell Polarization Programs and a Biphasic Regulatory Response
Source: Sci Rep. 2016 Nov 25;6:37790. doi: 10.1038/srep37790 (PMC5122918; doi:10.1038/srep37790)
Supplement: Supplementary Information [file srep37790-s1.pdf]

MC5r and A2Ar Deficiencies During Experimental Autoimmune Uveitis Identifies Distinct T cell Polarization Programs and a Biphasic Regulatory Response

Darren J. Lee[1*], Janine Preble[2,3], Stacey Lee[2,3], and C. Stephen Foster[2,3,4], Andrew W. Taylor[5]

[1]Department of Ophthalmology/Dean McGee Eye Institute, University of Oklahoma Health Sciences Center, Oklahoma City, Oklahoma, USA

[2]Massachusetts Eye Research and Surgery Institute, Waltham, Massachusetts, USA.
[3]Ocular Immunology and Uveitis Foundation, Waltham, Massachusetts, USA.
[4]Harvard Medical School, Boston, Massachusetts, USA

[5]Department of Ophthalmology, Boston University School of Medicine, Boston, Massachusetts, USA.

*Corresponding author
Darren J. Lee, PhD
Office: (405) 271-3642
Fax: (405) 271-8128
darren-lee@ouhsc.edu

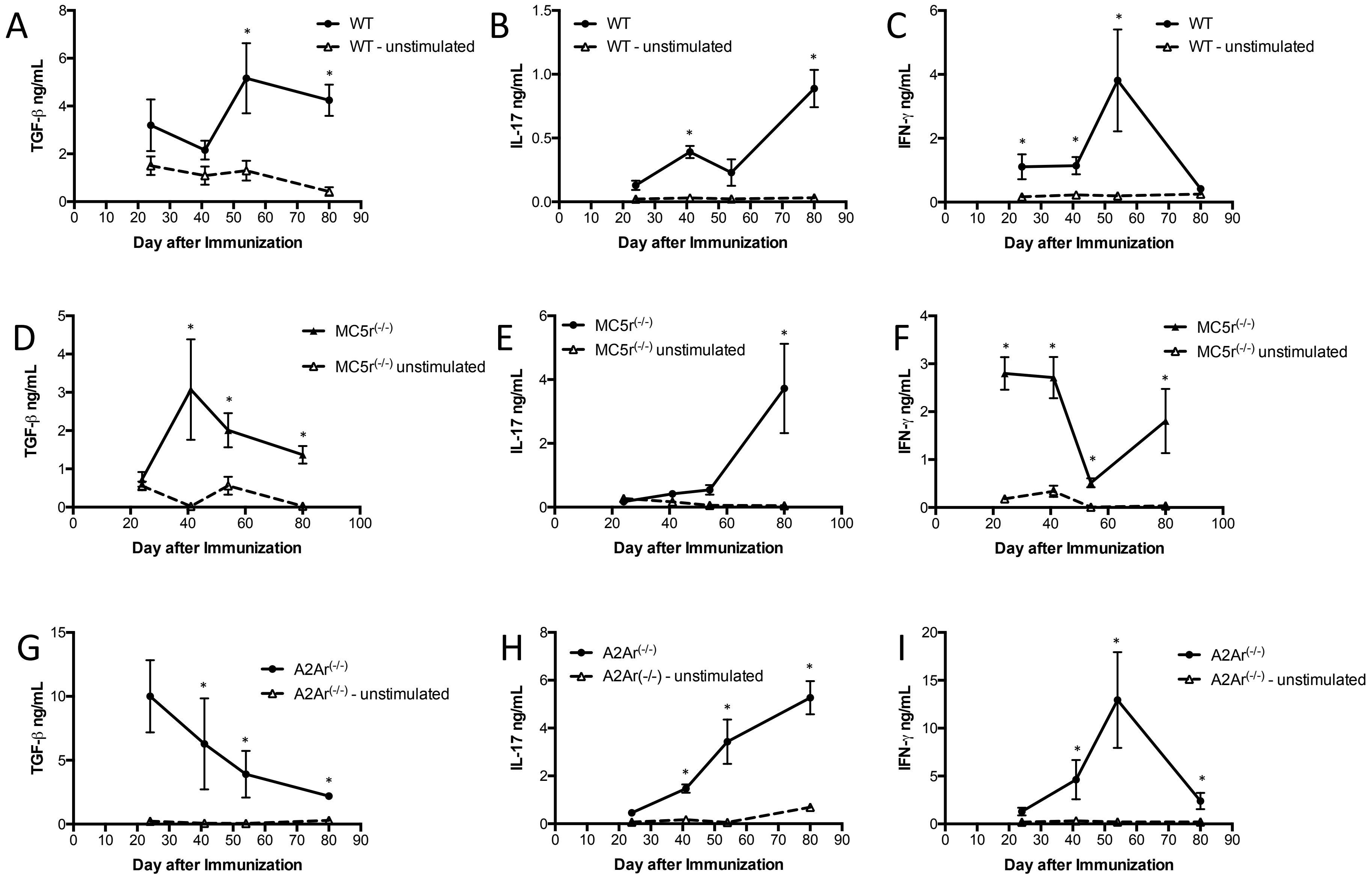

Supplementary Figure 1. Time course of cytokine profiles during EAU. Spleens from mice at day 24, 41, 54 and 80 during EAU were collected and cultured as in Figure 1 with and without IRBP. The dashed line and open triangles indicate the mean and SEM cytokine concentration of spleen cells cultures without IRBP. The mean and SEM of the cytokine concentrations of spleen cell cultures with IRBP from Figure 1 are depicted as the solid line and closed circle. The cytokine profile of wild-type mice (A-C), MC5r$^{(-/-)}$ mice (D-F), and A2Ar$^{(-/-)}$ mice (G-I). The TGF-b profile (A, D, G), IL-17 profile (B, E, H), and IFN-g profile (C, F, I). Each time point is an average ± SEM of 5-10 mice collected from at least three different experiments. Statistical significance ($P \leq 0.05$) is designated by *.

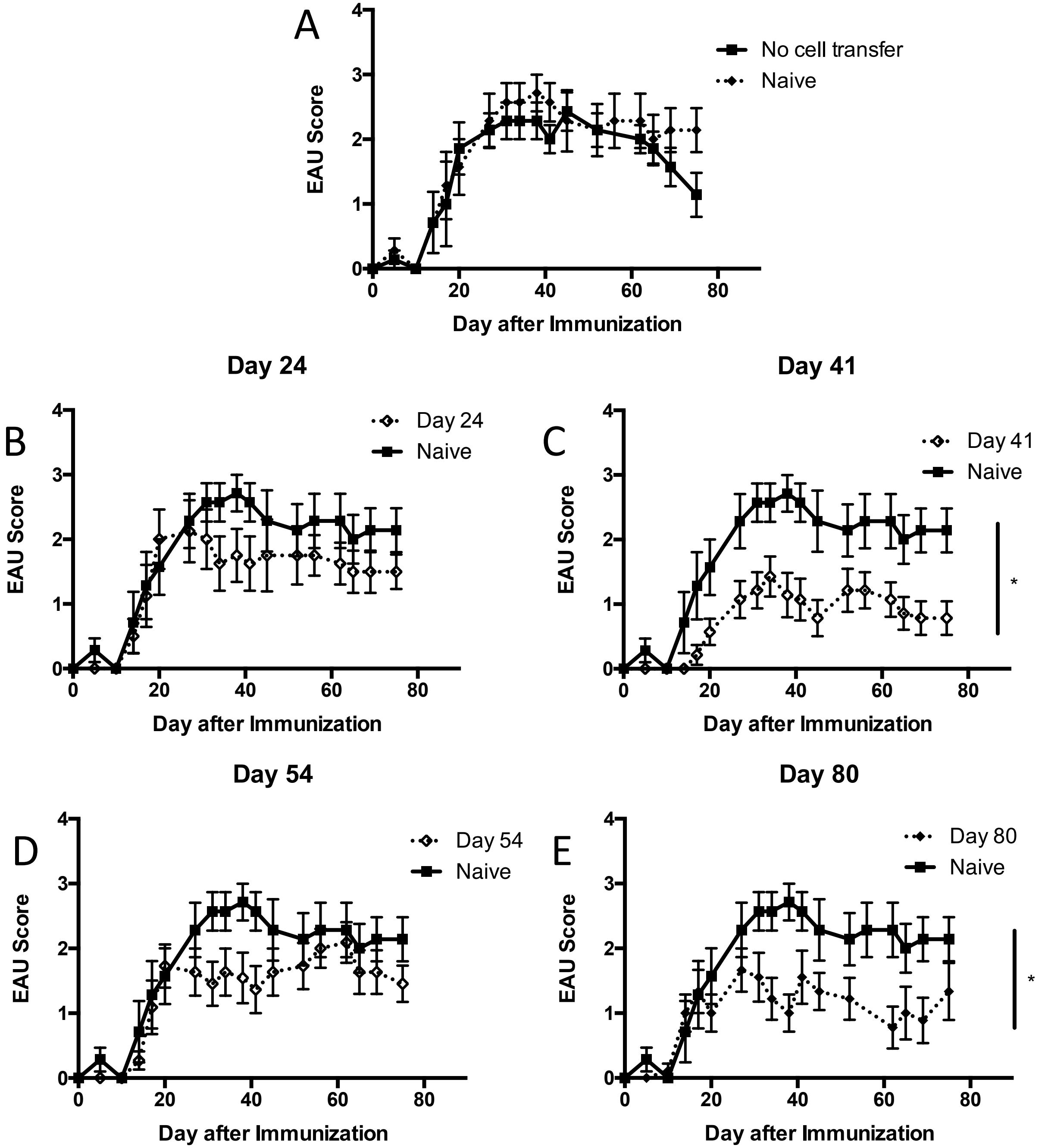

Supplementary Figure 2. Assay for functional regulatory activity in the spleen of EAU mice. The mean ± SEM of EAU scores from Figure 2 are shown as open circles and connected by a dashed line for reference. Donor control cells were from mice that were not immunized for EAU (naïve), but were aged to be equivalent to the EAU Day 80 mice. Shown are EAU scores of mice that did not receive any cells (solid line) and mice that received naïve T cells (dashed line) (A). In order for better comparison, shown are the graphs from Figure 2 of mice that received spleen cells from mice at Day 24, 41, 54, or 80 during EAU (B-E) as open circles and a dashed line compared with the mice that received naïve spleen cells (solid line). Each time point is an average ± SEM of 5-10 mice collected from at least three different experiments. Statistical significance ($P \leq 0.05$) is designated by *.

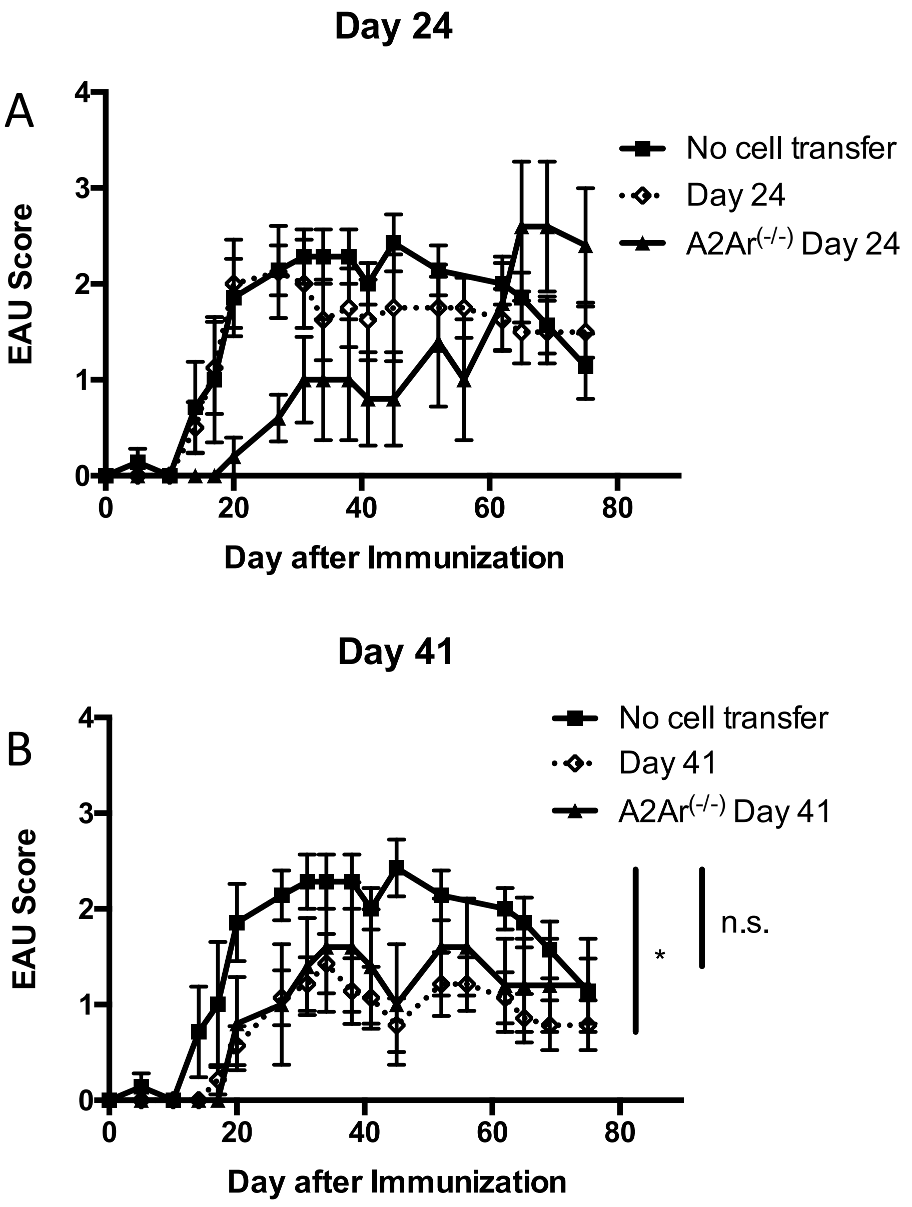

Supplementary Figure 3. Assay for functional regulatory activity in the spleen of A2Ar$^{(-/-)}$ EAU mice. Graphs from Figures 2 and 3 are combined for better comparison. Spleens from wild-type and A2Ar$^{(-/-)}$ mice immunized for EAU were collected at the onset (day 24) and chronic phase (day 41) of EAU. Spleen cells were re-activated in vitro with IRBP and transferred to recipient mice immunized for EAU. Shown are mean EAU scores ± SEM of each group of mice each day. The mice groups are mice that did not receive spleen cells (solid line), and the mice that did (dashed line) receive spleen cells from MC5r$^{(-/-)}$ mice at day 24 (A), or day 41 (B), or mice that received spleen cells from A2Ar $^{(-/-)}$ mice at day 24 (C), or day 41 (D). Each experiment was repeated 2-3 times and represents 6-12 recipient mice per group. Statistical significance ($P \leq 0.05$) is designated by *.

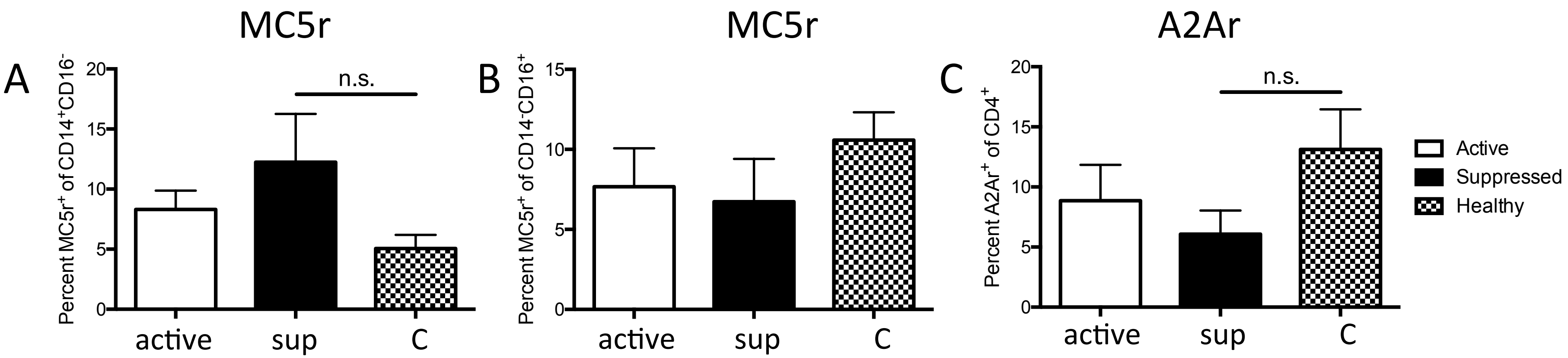

Supplementary Figure 4. Expression of MC5r and A2Ar on PBMC subsets from uveitis patients. Shown are bar graphs of the mean ± SEM of the percentage of cells that express the indicated receptor. Whole blood was collected from non-infectious uveitis patients or non-uveitis controls (C, n = 11). Uveitis patients from Fig that were in the active group (UA) defined as having inflammation within a year from the time of the blood draw are further subdivided into two groups based on the uveitis status at the time of collection. Patients with active inflammation at the time of collection (active, n = 11) or the suppressed patients have no inflammation within 2-7 months of the time of collection (supp, n = 9). PBMCs were isolated and stained for CD14, CD16, CD4, MC5r and A2Ar. Shown is the percentage of $CD14^+CD16^-$ cells that are $MC5r^+$ (A), percentage of $CD14^-CD16^+$ cells that express MC5r (B), and percentage of $CD4^+$ cells that express A2Ar. No statistical significance is designated by n.s.
